# Enhancing *DPYSL3* gene expression via a promoter-targeted small activating RNA approach suppresses cancer cell motility and metastasis

**DOI:** 10.18632/oncotarget.8290

**Published:** 2016-03-23

**Authors:** Changlin Li, Wencong Jiang, Qingting Hu, Long-cheng Li, Liang Dong, Ruibao Chen, Yinghong Zhang, Yuzhe Tang, J. Brantley Thrasher, Chang-Bai Liu, Benyi Li

**Affiliations:** ^1^ Department of Urology, University of Kansas Medical Center, Kansas City, KS 66160, USA; ^2^ Department of Urology, The Affiliated Hospital, Guangdong Medical University, Zhanjiang 524001, China; ^3^ Institute of Cell Therapy, China Three Gorges University, Yichang 443002, China; ^4^ Laboratory of Molecular Medicine, Peking Union Medical College Hospital, Chinese Academy of Medical Sciences, Beijing 100073, China

**Keywords:** DPYSL3, CRMP4, metastasis, RNAa, saRNA

## Abstract

To explore a novel strategy in suppressing tumor metastasis, we took the advantage of a recent RNA activation (RNAa) theory and used small double-strand RNA molecules, termed as small activating RNAs (saRNA) that are complimentary to target gene promoter, to enhance transcription of metastasis suppressor gene. The target gene in this study is Dihydro-pyrimidinase-like 3 (*DPYSL3*, protein name CRMP4), which was identified as a metastatic suppressor in prostate cancers. There are two transcriptional variants of *DPYSL3* gene in human genome, of which the variant 2 is the dominant transcript (DPYSL3v2, CRMP4a) but is also significantly down-regulated in primary prostate cancers. A total of 8 saRNAs for DPYSL3v1 and 14 saRNAs for DPYSL3v2 were tested in multiple prostate cancer cell lines. While none of the saRNAs significantly altered DPYSL3v1 expression, 4 saRNAs showed a strong enhancing effect on DPYSL3v2 expression, resulting in reduced cell mobility *in vitro*. To achieve a prostate cancer-specific delivery for *in vivo* testing, we conjugated the most potent *sa*V2-9 RNA molecule with the prostate-specific membrane antigen (PSMA)-targeting aptamer A10-3.2. The conjugates successful increased DPYSL3v2 gene expression in PSMA-positive but not PSMA-negative prostate cancer cells. In nude mice bearing orthotopic xenograft of prostate cancer, a 10-day consecutive treatment with the *sa*V2-9 conjugates significantly suppress distal metastasis compared to the control saRNAs. Analysis of xenograft tissues revealed that DPYSL3v2 expression was largely increased in *sa*V2-9 conjugate-treated group compared to the control group. In conclusion, DPYSL3v2 promoter-targeted saRNA molecules might be used as an adjunctive therapy to suppress prostate cancer metastasis.

## INTRODUCTION

Metastasis is the major cause of mortality from prostate cancer [1, 2] and systemic metastasis often occurs following local therapy failure in high-risk patients, including locally advanced (positive surgical margin) or high grade (Gleason sum score ≥ 8) tumors [3, 4]. Prostate cancers often migrate to extra-prostatic tissue and local lymph nodes before distant organ metastasis. The 10-year progression-free survival probabilities were 79% for organ-confined disease, but only 12% for disease with lymph node metastases. Therefore, suppressing cancer cell invasion at a very early stage would prevent the development of distal metastatic disease and slow down disease progression, improving patient quality of life for prostate cancers.

Dihydropyrimidinase-related protein 3 (*DPYSL3*), also called collapsin response mediator protein 4 (CRMP4), is a member of *DPYSL* gene family that encodes five cytosolic phospho-proteins involved in semaphorin/collapsin-induced cellular events [5]. *DPYSL* gene family (*DPYSL1-5*) shares about 50-70% sequence homology [6–9]. Previous studies indicated that CRMP1 protein is an invasion suppressor in human glioma and lung cancers [10–12], but CRMP-2 and CRMP-5 proteins were extensively expressed in colorectal cancers and high-grade lung neuro-endocrine carcinomas, respectively [12, 13]. In a search for metastasis-associated proteins using proteomics approach, we previously identified CRMP4 protein as a tumor metastasis suppressor in prostate cancers [14]. Once overexpressed or up-regulated in prostate cancer cells, CRMP4 protein suppressed cell motility or invasion *in vitro* and reduced tumor metastasis in mouse xenograft models [14–16]. Therefore, up-regulating *DPYSL3* gene expression in prostate cancer is expected to suppress tumor metastasis, providing a significant benefit for locally advanced high-risk prostate cancer patients.

Small double-strand activating RNA (saRNA) molecules that are complementary to the gene promoter region have been demonstrated to transcriptionally up-regulate target gene expression [17–19]. This phenomenon is termed as RNA activation (RNAa) and is evolutionarily conserved across species [20]. It has been shown that the saRNAs targeting the promoter region of tumor suppressor genes, such as E-cadherin, p21^cip1^ and Krüppel-like family of transcription factor-4, inhibited tumor cell growth *in vitro* and *in vivo* [21–24]. Thus, we hypothesized that saRNAs with optimal properties can be used to increase the expression of silenced tumor suppressor genes such as *DPYSL3* in prostate cancers.

In this study, we screened a series of saRNA molecules targeting *DPYSL3* gene promoter region and identified several saRNAs that could effectively enhance *DPYSL3* gene expression at the transcription level *via* a promoter-dependent mechanism. Transfection of these saRNAs into prostate cancer cells significantly reduced cancer cell migration and invasion *in vitro*. Injection of these saRNAs conjugated with a prostate cancer-homing aptamer molecule dramatically suppressed distal metastasis of prostate cancer in a mouse orthotopic xenograft model.

## RESULTS

### DPYSL3v2 gene expression is downregulated in metastatic prostate cancers

Human *DPYSL3* gene has two transcriptional variants due to distinct promoter usage [25], as illustrated in supplemental Figure S1. These two isoforms of *DPYSL3* gene encode two proteins that differ in their N-terminal amino acid sequence of exon 1 region [7, 25]. The isoform-1 has 2055 nt in cDNA nucleotide sequence while isoform-2 is 1713 nt. These isoforms are translated to proteins of CRMP4b (DPYSL3v1, 684 aa, 75 KD) and CRMP4a (DPYSL3v2, 570 aa, 64 KD).

**Figure 1:**
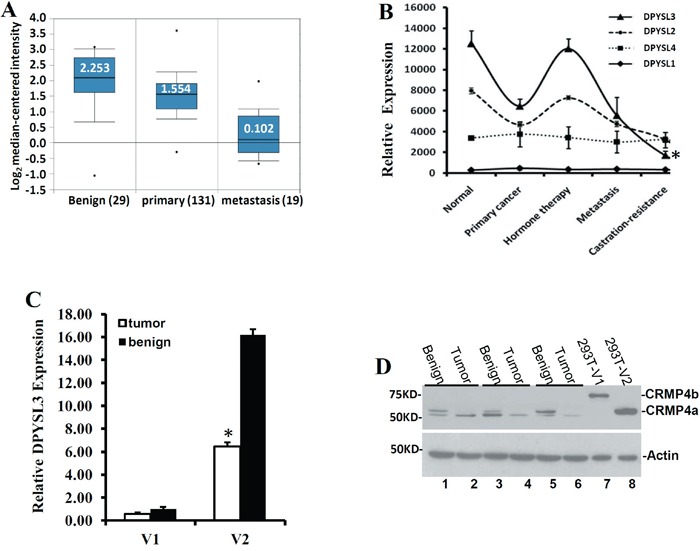
DPYSL3v2/CRMP4a expression is reduced in metastatic prostate cancers **A.** Public dataset [26] was extracted from Oncomine™ and graphed in to three groups. The numbers in white indicate the median value and patient case number is indicated on x-axel. **B.** cDNA microarray dataset from a previously published report [27] was re-analyzed. Data represent the Mean from different patient groups, including benign prostate specimens (normal, n = 5), primary cancers (n = 23), tumors after hormone therapy (n = 17), metastasis (n = 9) and castration-resistant tumors (n = 3). The errors bars indicate the standard error of mean (SEM). The asterisk indicates a significant difference compared to other groups (p < 0.05, student's *t*-test). **C.** Quantitative analysis of *DPYSL3* variants in prostate cancers were conducted using total RNAs extracted from frozen tumor specimens and the individually matched nonmalignant compartments, as described [46]. The expression levels of *DPYSL3* variants were normalized against the epithelium-specific gene KRT18 before the relative values were calculated. The relative ratio of gene expression level in malignant compared to benign tissues was presented as fold induction. Error bar represents the SEM. The asterisk indicates a significant difference compared to the ‘normal’ group (p < 0.05, student's *t*-test). **D.** Western blot assays were used to evaluate CRMP4 protein expression in 3 pairs of prostate specimens. Cell lysates from 293T cells overexpressing CRMP4a and CRMP4b were used to as positive control. Actin blot served as protein loading control. Data represent two separate experiments.

We examined the expression profiles of these two isoforms in human prostate cancers and prostate cancer cell lines. In the online database Oncomine™, 9 out of 14 published datasets showed a significant reduction of *DPYSL3* gene expression in malignant tissues compared to the benign tissues (Table 1) and the fold reduction was from 1.705 to 3.325. Analysis of one dataset from publically available Oncomine™ database [26] revealed that *DPYSL3* expression was largely reduced in metastatic prostate cancer tissues compared to benign prostatic tissues (about 20-fold) and primary prostate cancers (about 15-fold) (Figure 1A). We also re-analyzed a published cDNA microarray dataset generated from prostate cancer tissues as described previously [27, 28] and identified a clear association of *DPYSL3* gene reduction along with disease progression from primary cancer to castration-resistant metastatic cancers (Figure 1B). These data further confirm our previous report [14] that *DPYSL3* gene expression is reduced in metastatic prostate cancers.

**Table 1:** ONCOMINE™ database analysis of DPYSL3 gene expression

Reported studies	Cases		Fold change	*P*
	Normal prostate	Prostate Cancers		
**Welsh et al. Cancer Res 2001**	**9**	**25**	**−3.325**	**9.39E-5**
**Grasso et al. Nature 2012**	**28**	**59**	**−2.199**	**9.57E-9**
**Arredouani et al. Clin Cancer Res 2009**	**8**	**13**	**−2.118**	**5.24E-5**
**Yu et al. J Clin Oncol 2004**	**23**	**65**	**−2.070**	**1.83E-8**
**Lapointe et al. PNAS 2004**	**41**	**60**	**−1.888**	**1.87E-12**
**Vanaja et al. Cancer Res 2003**	**8**	**27**	**−1.867**	**2.74E-4**
**Liu et al. Cancer Res 2006**	**13**	**44**	**−1.850**	**1.24E-9**
**Luo et al. Mol Carcinog 2002**	**15**	**15**	**−1.808**	**1E-3**
**Taylor et al. Cancer Cell 2010**	**29**	**131**	**−1.705**	**2.148E-8**
LaTulippe et al. Cancer Res 2002	3	23	−2.776	0.058
Varambally et al. Cancer Cell 2005	6	7	−1.462	0.071
Magee et al. Cancer Res 2001	4	8	−1.588	0.084
Sing et al. Cancer Cell 2002	50	52	−1.193	0.314
Wallace TA et al. Cancer Res. 2008	20	69	−1.042	0.407

Expression profiles of *DPYSL3* genes in 14 cDNA microarray datasets were extracted from the Oncomine database along with the publication citations, the case numbers, fold induction and the P values. Bold fonts indicate data with statistical significance.

To understand if *DPYSL3* isoforms are differently expressed in prostate cancer tissues, we conducted a real-time PCR analysis of prostate tissues obtained from radical prostatectomy. Quantitative data revealed that DPYSL3v2 transcript was the dominant one with a remarkably higher level than DPYSL3v1 transcript. However, DPYSL3v2 levels were significantly lower in malignant tissues compared to that in case-matched surrounding benign tissues (Figure 1C). These results were consistent with our previous report [14]. At the protein level, only CRMP4a (encoded by DPYSL3v2) but not CRMP4b (encoded by DPYSL3v1) was detected in both benign and malignant tissues (Figure 1D), which is consistent with the low mRNA expression level of DPYSL3v1 gene transcript in prostate tissues. Nonetheless, CRMP4a protein levels were much lower in malignant tissues compared to their benign counterparts, similar to the mRNA expression pattern. Interestingly, CRMP4a protein exerted as a duplet band in benign tissues but as a single band in malignant tissues, indicating a protein modification that is lost in malignant tissue, for example, CRMP4 was reported to be phosphorylated by GSK-3 after CDK5/DYRK2 prime phosphorylation [29].

### Promoter-targeted saRNA enhances DPYSL3v2 gene expression

**Figure 2:**
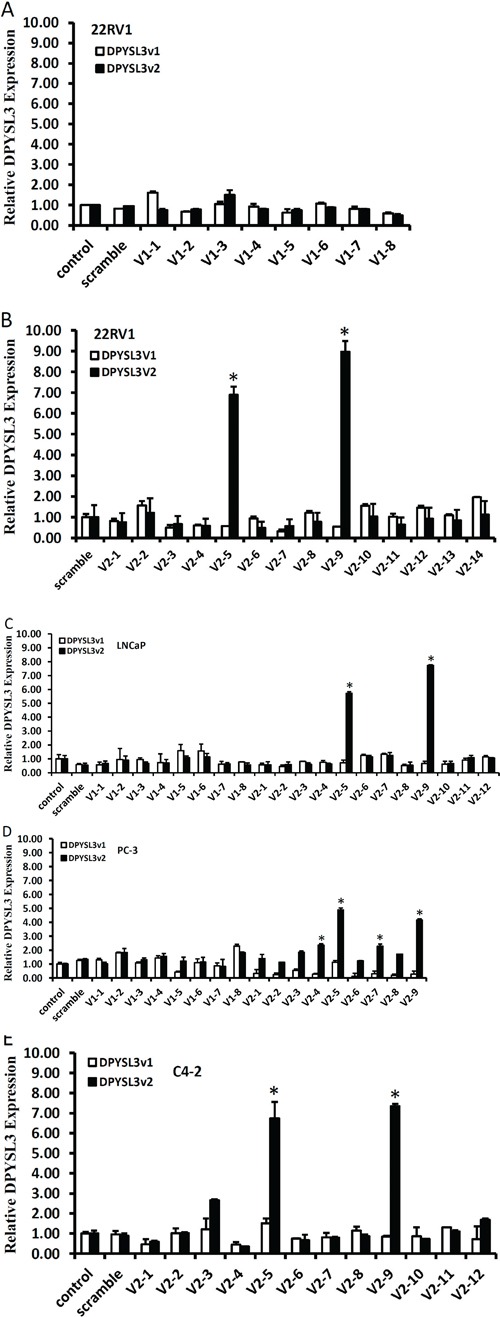
Promoter-targeting saRNAs enhance DPYSL3v2 expression in prostate cancer cells A total of 8 saRNAs for DPYSL3v1 promoter and 14 saRNAs for DPYSL3v2 were transfected into prostate cancer 22RV1 panel **A** and **B.** LNCaP **C.** PC-3 **D.** and C4-2 **E.** as indicated at a final concentration of 10 nM in media in 6-well plates. Cells were harvested 3 days later for qPCR assays. After normalized with S18 gene expression levels, fold induction against the mock control transfection (set of 1) was calculated and graphed. Data were shown as the average from three independent experiments and the error bar represents the SEM. The asterisk indicates a significant difference compared to the control (p < 0.05, student's *t*-test).

To enhance DPYSL3 gene expression, we utilized a recently invented small activating RNA (saRNA) approach. Multiple saRNA molecules were designed to target *DPYSL3* promoters based on the criteria reported previously [17, 20]. The saRNA targeting sites were shown in Figure S2A and S2B, and their sense DNA sequences were listed in Table 2. Four different prostate cancer cell lines were used in screening active saRNAs with quantitative real-time PCR assays. A total of 8 saRNAs targeting the DPYSL3v1 promoter was tested but none of them had any significant enhancing effect on DPYSL3v1 expression (Figure 2A-2E). In contrast, 4 out of 14 saRNAs targeting the DPYSL3v2 promoter actively enhanced gene expression in different cell lines, especially two (*sa*V2-5 and *sa*V2-9) of these 4 saRNAs exerted dramatic enhancing effect in all cell lines tested (Figure 2A-2E). There was no cross effect observed between the promoter-specific saRNAs.

**Table 2:** *DPYSL3* saRNA target sequence and genomic location on Chromosome 5

saRNA#	start	end	sense	promoter site
V1-1	147510470	147510452	GGATTGTGAGTCTGTCGTA	V1p-414/-396
V1-2	147510652	147510634	GGGCTTTTGTGACTCTCAA	V1p-596/-578
V1-3	147510738	147510720	GTGATCTGAAATGAGGCAA	V1p-698/-664
V1-4	147510793	147510775	GAGTGCTTTGCAAGGCAAA	V1p-737/-719
V1-5	147510816	147510798	ATGAGGGACAGAGCATCAA	V1p-760/-742
V1-6	147510852	147510834	TGTCAGTGTGTGAGATTAA	V1p-796/-778
V1-7	147510853	147510835	GTGTCAGTGTGTGAGATTA	V1p-797/-779
V1-8	147510909	147510891	ATCTGGGCTTAGGTGTGAA	V1p-853/-835
V2-1	147453872	147453854	AGCTGGCGCAGCAAAAGAA	V2p-178/-160
V2-2	147453945	147453927	GGAAAATCAATAGGGATAA	V2p-248/-230
V2-3	147453958	147453940	GCAGATGCAAAGAGGAAAA	V2p-261/-243
V2-4	147453959	147453941	GGCAGATGCAAAGAGGAAA	V2p-262/-244
**V2-5**	**147453960**	**147453942**	**AGGCAGATGCAAAGAGGAA**	**V2p-263/-245**
V2-6	147454014	147453996	CAGCACTCGCGAATCAGAA	V2p-317/-299
V2-7	147454430	147454412	CACGGCTTTCCATTTTCTA	V2p-733/3715
V2-8	147454534	147454516	AGGCAGTGGAGTTTCTTTA	V2p-837/-819
**V2-9**	**147454573**	**147454555**	**GCAGCATTCATGTTCTTTC**	**V2p-876/-858**
V2-10	147454717	147454699	ACTCGGGATTGTGAGCAGT	V2p-1022/-1002
V2-11	147454722	147454704	AGTAAACTCGGGATTGTGA	V2p-1027/-1007
V2-12	147454795	147454777	TGTATTCCATCGCCGAAGG	V2p-1100-1080
V2-13	147455020	147455002	GATTGACAATTGGGGAGCG	V2p-1325/-1305
V2-14	147455219	147455201	AGCCCAAGCCGGAGTATTC	V2p-1524/-1504

**Figure 3:**
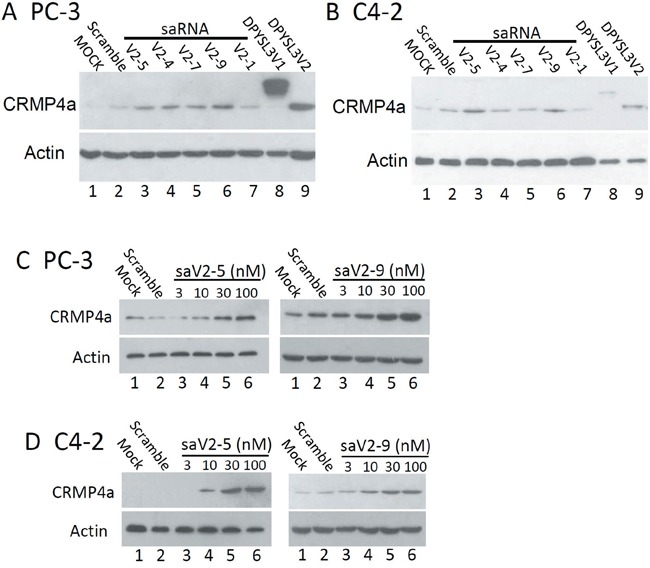
Promoter-targeting saRNAs enhance CRMP4a expression in prostate cancer cells Selective saRNAs were transfected into PC-3 or C4-2 cells at a concentration of 10 nM **A** and **B.** or as indicated in each panel **C** and **D.** for 3 days, and cell lysates were subjected to western blot assays for CRMP4a expression. The scramble control saRNAs were used at 10 nM. Cellular protein lysates from PC-3 or C4-2 cells over-expressing exogenous CRMP4a or CRMP4b were used as positive control. Actin blot served as protein loading control.

We then evaluated the saRNAs on gene expression at the protein level. Based on the qPCR data, we chose five saRNAs, including the non-effective *sa*V2-1, less effective *sa*V2-4 and *sa*V2-7, plus the most effective *sa*V2-5 and *sa*V2-9. Western blot data showed a consistent upregulation of CRMP4a protein levels after transfection of the saRNAs in a similar order as seen in qPCR assays (Figure 3A-3B). In detail, *sa*V2-5 and *sa*V2-9 exerted a stronger effect on CRMP4a protein expression compared to other saRNAs. They also exerted a dramatic dose-dependent enhancing effect on CRMP4a protein expression (Figure 3C-3D). These data indicate that *sa*V2-5 and *sa*V2-9 saRNAs are potent enhancers in upregulating *DPYSL3* gene expression.

**Figure 4:**
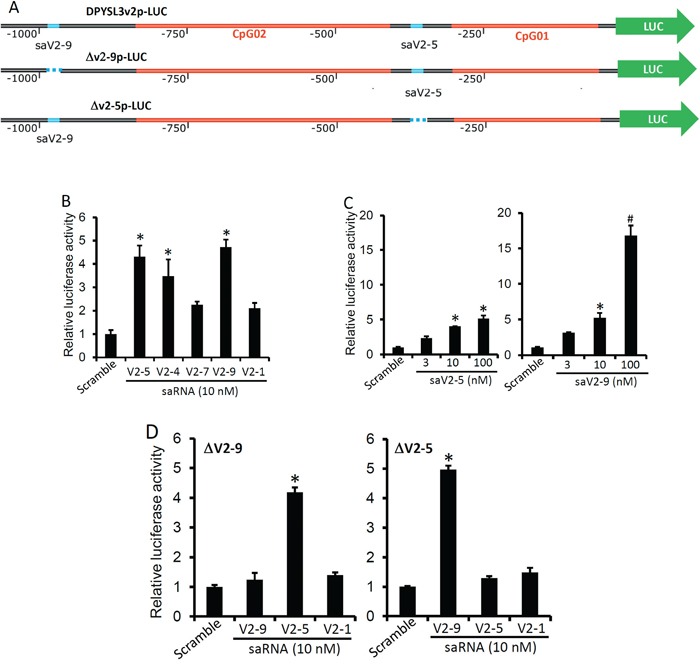
Promoter-specific effect of the saRNAs on DPYSL3 expression **A.** Scheme of DPYSL3v2 promoter-driven luciferase reporters. Δv2-9 or Δv2-5 indicates a fragment deletion of the saRNA-targeted site. B-D PC-3 cells plated in 6-well plates were infected with lentiviral particle containing the parental reporter (B & C) or individual mutant reporter (D) and then transfected with the saRNAs at a dose of 10 nM **B.** or as indicated in the panel **C** and **D.**. The scramble saRNA was used at 10 nM. Luciferase activity was measured one day later and the fold induction was calculated against the scramble control. Data were shown as the average and the error bar represents the SEM. The asterisk indicates a significant difference compared to the control (p < 0.05, student's *t*-test).

To further examine if saRNA-enhanced *DPYSL3* gene expression is a promoter-specific activity, we created a luciferase reporter construct driven by a 1250-bp segment (−1275/−25 from the transcription start site) of DPYSL3v2 promoter, as illustrated in Figure 4A. The reporters were packaged as lentiviral particles. Transfection of the saRNAs in PC-3 cells infected with the lentiviral luciferase reporter (DPYSL3v2p-LUC) significantly enhanced the reporter activity (Figure 4B), of which the *sa*V2-9 saRNA displayed the most potent effect compared to others (Figure 4C). To confirm the sequence specificity of the saRNAs, we generated two mutant reporters by deleting the targeting segments of *sa*V2-5 (Δv2-5p-LUC) and *sa*V2-9 (Δv2-9l-LUC) saRNAs on the promoter individually (Figure 4A). As expected, deleting the corresponding segment on the promoter abolished the saRNA-induced reporter activity (Figure 4D). These data demonstrate that saRNA-mediated gene activation is indeed a promoter-specific event of transcriptional activation.

### *DPYSL3* saRNAs suppress cancer cell migration and invasion

**Figure 5:**
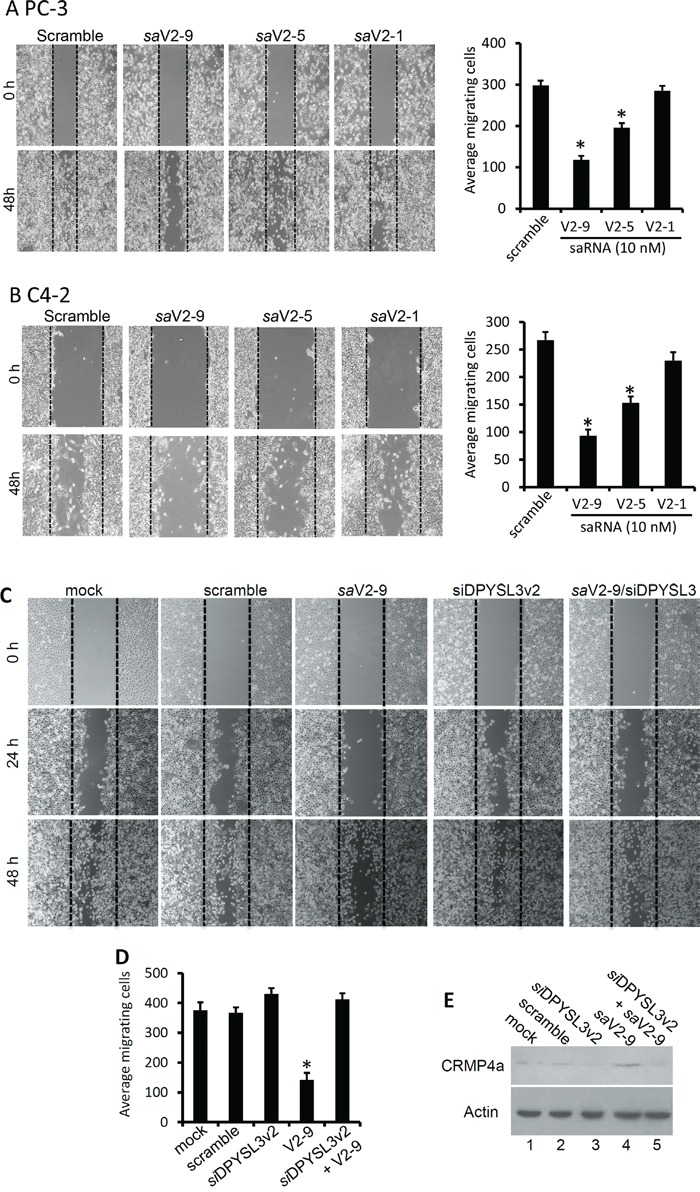
DPYSL3v2-targeted saRNAs suppress cell migration **A** and **B.** Cells as indicated were seeded in 24-well plates and transfected with the saRNAs as indicated (10 nM). A wound scratch was made with a sterile 200-μl pipet tip and cells migrated into the wound space were counted at day 3 and the representing microscopic fields were recorded (A-B). The quantitative data of migrated cells from 3 microscopic fields were summarized in the graph panels next to the image panel. **C** and **D.** Similar wound healing assay was conducted except a siRNA against DPYSL3v2 was transfected with or without the saV2-9 saRNAs. Microscopic images in panel C were taken from each treatment and the quantitative data from 3 microscopic fields were graphed in panel D. All small RNAs were used at a concentration of 10 nM in the media. **E.** CRMP4a protein levels in the experiments of panel C were evaluated by western blot assay. Actin blot served as protein loading control.

To assess the potential of DPYSL3v2 saRNAs as anti-metastasis agents, we then tested their effect on tumor cell migration and invasion *in vitro*. Cell migration was assessed with a wound healing assay. As shown in Figure 5A-5B, transfection of *sa*V2-9 molecules significantly suppressed cell migration in both PC-3 and C4-2 cells compared to the scramble and the negative *sa*V2-1 saRNAs, while the *sa*V2-5 also significantly suppressed cancer cell migration at a less extent.

To verify if saRNA-mediated *DPYSL3* gene upregulation played a critical role in suppressing cancer cell migration, we co-transfected the *sa*V2-9 saRNA together with a small interfering RNA (siRNA) of *DPYSL3* gene in PC-3 cells. Comparing to the *sa*V2-9 alone that significantly reduced cell migration, co-transfection of *DPYSL3* siRNAs abolished *sa*V2-9-induced suppressing effect on cell migration (Figure 5C-5D). Notably, *DPYSL3* siRNA only had a slight enhancing effect on cell migration, possibly due to a lower level of the endogenous gene expressed in PC-3 cells (Figure 5E). These data suggest that DPYSL3v2 gene upregulation is a critical event in *sa*V2-9-mediated suppression on cell migration.

**Figure 6:**
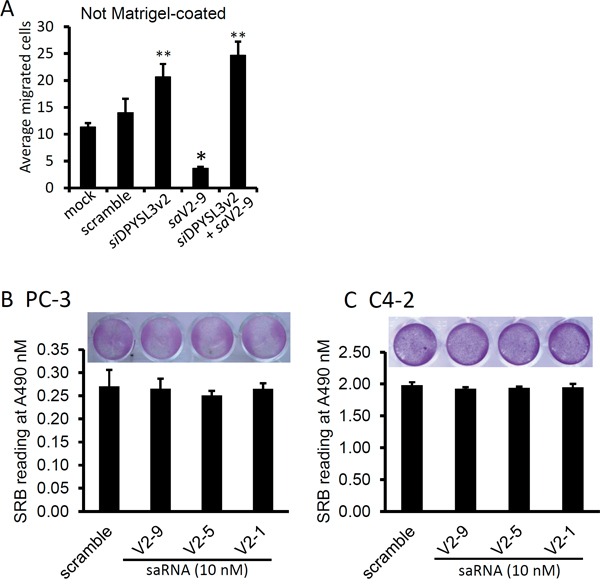
DPYSL3v2-targeted saRNAs suppress cell invasion **A.** After transfection with the saRNAs or siRNA at 10 nM concentration as indicated, PC-3 cells were loaded onto the upper chamber. Three days later the migrated cells were stained with Hoechst 33342 (0.5 μg/ml) and counted (Mean ± SEM). The asterisks indicate a significant difference compared to the mock control (* p < 0.01, ** p < 0.05, student's *t*-test). **B** and **C.** PC-3 or C4-2 cells were plated in 24-well plates and transfected with the saRNAs at indicated for 3 days. Cell growth was evaluated with SBR assay as described in the text. Representing microscopic images were inserted in the panels.

To assess the anti-invasion effect of the saRNAs, we utilized the Boyden-Chamber assay. As shown in Figure 6A, *sa*V2-9 transfection significantly reduced the numbers of migrated PC-3 cells compared to the scramble saRNA, in contrast, silencing DPYSL3v2 expression with siRNA approach largely increased PC-3 migration into low chamber. Consistently, co-transfection of the *sa*V2-9 with *DPYSL3* siRNA abolished the *sa*V2-9-mediated suppression but increased cell migration. To rule out the possibility that these anti-migration and anti-invasion effect was due to reduced overall cell growth, we tested the effect of saRNAs on cell growth in a sulforhodamine B (SRB) assay [30]. As shown in Figure 6B-6C, the saRNAs used in the assays, either active or inactive on *DPYSL3* gene expression, had no significant effect on cell growth compared to the scramble saRNA in both PC-3 and C4-2 cells. Taken together, these data demonstrate that the *sa*V2-9 molecule is capable in suppressing cell migration by potently up-regulating DPYSL3v2 gene expression in prostate cancer cells.

### *DPYSL3* saRNAs suppress tumor metastasis in orthotopic xenograft model

**Figure 7:**
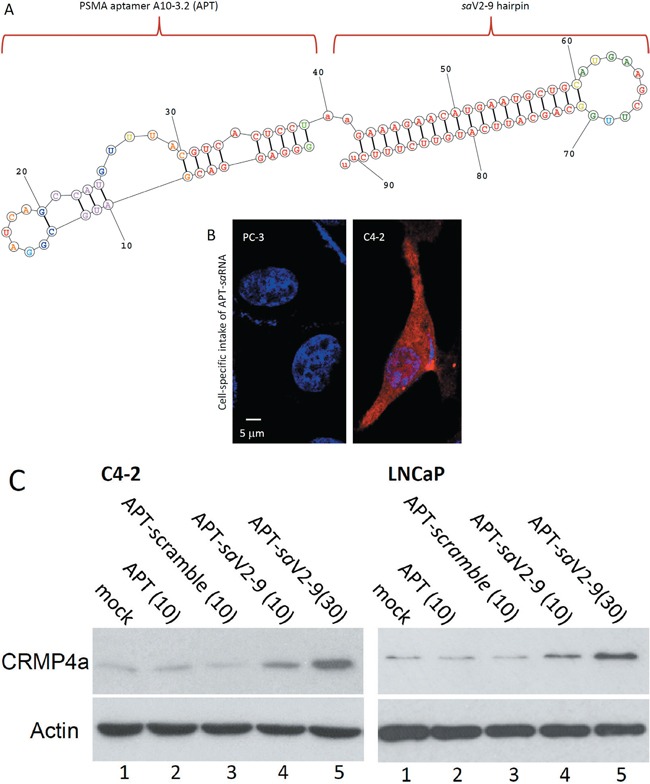
PSMA aptamer-conjugated saV2-9 enhances CRMP4a expression **A.** Scheme of the RNA conjugate of PSMA aptamer A10-3.2 with saV2-9 hairpin. Predicted RNA folding was performed online at http://rna.urmc.rochester.edu/RNAstructureWeb/. **B.** PC-3 or C4-2 cells were seeded in a chambered glass slide and the A10-3.2-V2-9 conjugates that were 5′-labeled with a fluorescent dye TYE™ 563 were added into cell culture at 10 nM for 30 min. After nuclear staining with Hoechst 33342, cells were evaluated under a confocal fluorescent microscope for cellular intake of the conjugate molecules. **C.** C4-2 and LNCaP cells were seeded in 6-well plates and the RNA conjugates were added into cell culture for 3 days as indicated (concentration at nM). Cells were harvested for western blot assays with anti-CRMP4 antibodies. Actin blot served as protein loading control. Data represent two separate experiments.

To validate the *sa*V2-9 as a metastasis inhibitor *in vivo*, we conducted a proof-of-concept experiment in nude mice bearing orthotopic prostate xenografts derived from C4-2 cells. First, to achieve a prostate cancer cell-specific delivery of the saRNA molecule, we conjugated the small hairpin-structured *sa*V2-9 RNA molecule with the prostate cancer-specific RNA aptamer A10-3.2 (Figure 7A). A10-3.2 is a specific ligand for prostate-specific membrane antigen (PSMA) [31, 32]. PSMA-dependent cellular intake was confirmed with a fluorescent-labeled APT-*sa*V2-9 conjugate in PSMA-positive C4-2 but not in PSMA-negative PC-3 cells (Figure 7B). Western blot assays demonstrated that this A10-3.2-conjugated *sa*V2-9 drastically enhanced CRMP4a protein expression in PSMA-positive C4-2 and LNCaP cells (Figure 7C).

**Figure 8:**
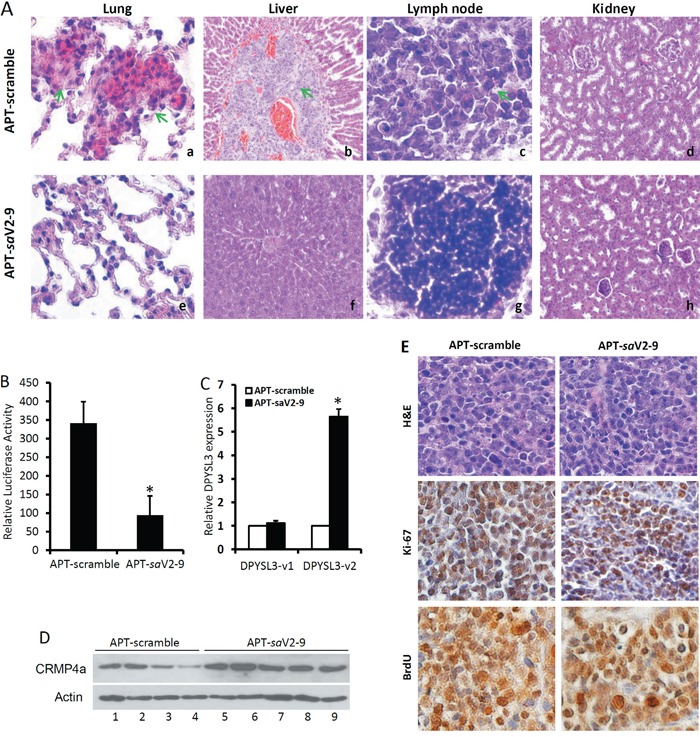
A10-3.2-saV2-9 conjugates suppress xenograft tumor metastasis *in vivo* **A.** Representing H&E section images from major organs as indicated harvested from animals received the APT-saV2-9 or the scramble control. The green arrows indicate metastatic cancer cells in left lung (a), liver (b) and lymph node (c) sections from animals received the control saRNA. No metastatic cancer cells were seen in kidney sections or other organs. **B.** The whole right lobes of lung harvested from animals treated with the scramble or *sa*V2-9 conjugates were homogenized and protein extracts were collected for luciferase activity measurement. The readings were normalized with the corresponding protein concentrations expressed as relative LUC reading/mg lung proteins. Data presented are MEAN ± SEM and the asterisk indicates a significant difference compared to the scramble control (P < 0.05, Student's t-test). **C.** Total RNAs were extracted from xenograft tissues and subjected to qPCR analysis for DPYSl3 gene expression. After normalized with KRT18 gene expression levels, relative fold induction against the value in the samples from the scramble control group was calculated and presented as MEAN ± SEM. The asterisk indicates a significant difference compared to the scramble control (p < 0.05, student's *t*-test). **D.** Total proteins were extracted from xenograft tumors and subjected to western blot assays for CRMP4a protein expression. Actin blot served as protein loading control. **E.** Xenograft tumors obtained from animals treated with the scramble or saV2-9 conjugates were processed for H&E staining, Ki-67 and BrdU immunohistochemical staining. Representing microscopic images were taken from a 200x magnification.

Next, we evaluated the anti-metastasis potential of the A10-3.2-*sa*V2-9 conjugates in mice bearing orthotopic C4-2 xenografts. A scramble RNA molecule conjugated with the A10-3.2 aptamer was used as the negative control. C4-2 cells were stably infected with lentiviral CMV-LUC reporter to express luciferase for *in vivo* imaging. Once the orthotopic tumor was established as monitored by living animal imaging (IVIS system) at about 4 weeks after tumor cell inoculation in dorsal lobe of the prostate as described [14], animals were treated daily by intraperitoneal injection of the conjugates at 1.0 nM per treatment for 10 consecutive days. At the end of treatment, animals were sacrificed and xenograft tumors, major organs and reginal lymph nodes were harvested for further analyses. Histological evaluation of major organs was conducted to determine regional and distal metastasis. As summarized in Table 3, there were no significant differences in animal body weight and the wet weight of orthotopic xenografts between the scramble control and *sa*V2-9 groups. Animals received the scramble saRNA treatment all displayed metastatic lessons at regional lymph nodes and lung. One of the animals even had a big liver mass as a metastatic tumor (Figure 8A). However, animals received the *sa*V2-9 treatment only had one incidence of reginal (lymph node) but not distal (lung) metastasis as evaluated histologically on H&E sections (Figure 8A). No sign of tumor metastasis in kidney.

**Table 3:** DPYSL3 saRNA suppresses tumor metastasis *in vivo*

parameters	APT-scramble	APT-*sa*V2-9	p value
body weight (gm ± SEM)	32.3 ± 1.4	31.8 ± 1.7	n.s.
O.T. tumor weight (gm ± SEM)	2.56 ± 0.87	2.71 ± 0.57	n.s.
lymph node met cases (%)	5/5 (100%)	1/5 (20%)	< 0.05
Lung/Liver met cases (%)	5/5 (100%)	0/5 (0%)	< 0.05

Because it is not easy to identify all micro-metastasis foci with conventional histological method, we performed a luciferase measurement of lung tissue protein extracts to determine total metastatic C4-2-LUC cells, as described recently [33]. As shown in Figure 8B, the luciferase activity in whole right lobe of lung tissues from *sa*V2-9 treated animals was significantly lower than that in the control group, indicating a dramatic suppression of lung metastasis.

We also examined *DPYSL3* gene expression in xenograft tissues. As expected, DPYSL3v2 (not DPYSL3v1) gene expression at the mRNA (Figure 8C) and CRMP4a protein (Figure 8D) levels were largely elevated in orthotopic xenograft tissues received *sa*V2-9 treatment compared to that from the control group. Consistent with cell growth data (Figure 6B-6C), cell proliferation markers (Ki-67 and BrdU labeling) remained similar in orthotopic xenograft tissues from these two treatments (Figure 8E). These data strongly suggest that the saRNA approach is a potent way to enhance endogenous gene expression *in vivo* and that prostate cancer cell-specific delivery of DPYSL3v2 promoter-targeting *sa*RNA is feasible in suppressing tumor metastasis in prostate cancer model.

## DISCUSSION

As a *proof-of-concept* project, this study demonstrated that the saRNA approach is a potent method to enhance the expression of tumor metastasis suppressor *DPYSL3* gene, which depends on the sequence specificity of the target gene promoter. Most importantly, this study provided the feasibility of *in vivo* application of *sa*V2-9 RNA to suppress tumor metastasis.

The association of reduced CRMP4a (DPYSL3v2 gene) protein level with prostate cancer metastasis was first established in screening metastasis-associated proteins using proteomic approach [14]. Further investigation confirmed *DPYSL3* gene/CRMP4 protein as a tumor metastasis suppressor because CRMP4a overexpression led to suppressed tumor cell motility *in vitro* and metastasis *in vivo* of prostate cancer cells [14]. In this study, we provided further evidences that *DPYSL3* gene expression (variant 2 only) is significantly lower in primary prostate cancers than that in benign tissues and that CRMP4a protein levels are largely reduced in prostate cancers compared to the surrounding benign tissues. In addition, a potential alteration on CRMP4a protein modification is evidenced in malignant tissues compared to benign tissues, depending on further analysis. In contrast, DPYSL3v1 gene expression in prostate tissues is very low at the mRNA level and its coding protein is missing or undetectable.

Prevention of metastasis has been a major effort to reduce cancer mortality [34]. In attempt to develop a novel anti-metastasis therapy for prostate cancer, we utilized the newly established saRNA technique to up-regulate the expression of tumor metastasis suppressor *DPYSL3* gene. Our data revealed that only DPYSL3v2 but not DPYSL3v1 gene is responding to the saRNA-mediated gene activation. In a total of 14 DPYSL3v2 promoter-targeted saRNAs, 4 of them showed a significant up-regulation of DPYSL3v2 gene expression at variable levels in multiple prostate cancer cell lines. The targeting sites of the most potent saRNAs on the promoter region are within 1kb range up-stream of the transcription start site and off the CpG island, consistent to published design criteria [17, 20]. These saRNAs induced a significant suppression of cell motility in multiple assays. Most significantly, delivery of the hairpin-structured saRNAs to prostate cancer cells *via* a PSMA aptamer largely suppressed tumor metastasis in an orthotopic xenograft model of prostate cancer. These findings are supported by a recent report using a TALE-dependent approach [15], in which promoter-specific demethylation caused a significant up-regulation of *DPYSL3* gene/CRMP4 protein expression, leading to reduced cancer cell migration and tumor metastasis. Taken together, it is conceivable that targeting *DPYSL3* gene promoter with our saRNA approach or the TALE-dependent demethylation is feasible to enhance endogenous DPYSL3v2 gene expression, resulting in increased CRMP4a protein levels *in vivo* and subsequent suppression of tumor metastasis. However, our saRNA approach possesses much more drug-like properties, including easy synthesis and delivery, and potential tissue-specific targeting. These saRNA molecules might be used as adjunctive therapy to conventional cytotoxic drugs in prostate cancer management, although the mechanism of CRMP4a-mediated suppression of tumor metastasis requires further investigation.

Successful delivery of therapeutic agents efficiently and specifically to the target tissue is desirable for treatment of human cancers [35]. Prostate specific membrane antigen (PSMA) is a well-known tumor antigen [36]. It is primarily expressed on the surface of prostate cancer epithelial cells and also is highly expressed in metastatic prostate cancer cells and the microvasculature of most studied tumors. This restricted expression pattern warranted it as a promising target for the diagnosis, detection, and management of prostate cancer. As such, PSMA has currently been used for molecular imaging, cancer vaccine development and targeted drug delivery in prostate cancers [37]. Particularly, an RNA-based aptamer targeting human PSMA was developed, which interacts specifically with the PSMA extra-cellular domain and has been widely used in targeted drug delivery and molecular imaging [31]. Recent reports defined two truncated PSMA aptamers with only 39-41mer in length but retraining the PSMA binding function [19, 32]. These truncated PSMA Aptamers make the chemical synthesis much easier to carry out and less non-specific binding [38]. In this study, we created a chimeric RNA molecule that the truncated A10-3.2 aptamer is conjugated to a hairpin-structured saRNA sequence. Consistent to previous reports from our group and others [32, 37, 39–41], A10-3.2 aptamer exerted a clean PSMA-specific cellular intake of the saRNA molecules in prostate cancer cells.

In conclusion, we identified 4 saRNA molecules that are potent in enhancing DPYSL3v2 gene expression *in vitro* and confirmed one of them is potent in suppressing tumor metastasis *in vivo*. The saRNA approach by targeting tumor suppressor gene such as *DPYSL3* represents a novel direction of cancer gene modulation and drug development.

## MATERIALS AND METHODS

### Cell lines, antibodies and reagents

Prostate cancer cell LNCaP, C4-2, 22RV1 and PC-3 lines were described in our previous publications [39, 40, 42] and maintained in a humidified atmosphere of 5% CO_2_, RPMI-1640 media supplemented with 10% fetal bovine serum (FBS) and antibiotics. The CMV-driven luciferase reporter stable PC-3 and C4-2 sub-lines were established using the pLenti CMV-Puro-LUC (w168-1) construct [43], a gift from Dr Eric Campeau (Addgene plasmid # 17477). All luciferase reporters were packaged in 293T cells with a lentiviral packaging psPAX2/pMD2.G system obtained from Dr Didier Trono as a gift (Addgene plasmid #12259-12260). Human embryonic kidney cell 293T line was purchased from American Type Culture Collection (ATCC, Manassas, VA) and cultured in DMEM media with 10% FBS and antibiotics. Antibodies for CRMP4 (sc-100323), Actin (sc-1616), Ki-67 (sc-23900) and DPYSL3 siRNA (sc-44487) were purchased from Santa Cruz Biotech (Santa Cruz, CA).

### Small activating RNA design, Aptamer-saRNA conjugate synthesis and transfection

DPYSL3 gene and promoter information on chromosome 5 was extracted on UCSD genome browser (https://genome.ucsc.edu/) and illustrated in supplemental Figure S1. Small activating RNAs were designed based on published protocols and criteria as described previously [20]. All saRNA sense targeting sequences were listed in Table 1 with information of chromosome location and relative distance from the transcription start site. Their locations on the promoter are illustrated in supplemental Figure S2. CpG islands were predicted using an online MethPrimer program (http://www.urogene.org/methprimer/). RNA duplexes including a fluorescence TYE™ 563-labeled APT-V2-9 molecule were chemically synthesized by IDT (Coralville, IA). Transfection was conducted with RNAiMAX obtained from Life Technologies (Carlsbad, CA).

PSMA aptamer A10-3.2 conjugate with the scramble and *sa*V2-9 saRNAs were synthesized with DuraScribe® T7 Transcription Kit from Epicentre (Madison, WI) based on the manufacturer's protocol as well as a previous report [32]. The DNA templates and primers were listed as follow: A10-3.2-scramble saRNA: TAA TAC GAC TCA CTA TAG GGA GGA CGA TGC GGA TCA GCC ATG TTT ACG TCA CTC CTA tct act gtc act cag tag t **ATGAAGC TTG** a cta ctg agt gac agt aga dTdT, A10-3.2-saV2-9: TAA TAC GAC TCA CTA TAG GGA GGA CGA TGC GGA TCA GCC ATG TTT ACG TCA CTC CTA gaa aga aca tga atg ctg c **ATGAAGCTTG** g cag cat tca tgt tct ttc dTdT. PSMA aptamer sequence is in upper case, the saRNA is in lower case and the hairpin loop is in bold upper case. The primers for *in vitro* T7-based synthesis are listed as follow: Aptamer-F: TAA TAC GAC TCA CTA TAG GGA GGA CG; Aptamer-R: AAT AGG AGT GAC GTA AAC ATG GCT; A1032-V2-9R: AAG AAA GAA CAT GAA TGC TGC C; A1032-scramble-R: AAT CTA CTG TCA CTC AGT AGT CAA GCT T. *In vitro* transcriptions were performed with modified T7 (Y639F) polymerase and nucleotides as described [32, 44].

### Plasmids, promoter cloning, luciferase reporter construction and luciferase detection

Plasmid constructs for human CRMP4a and CRMP4b expression in the pcDNA3.1-V5 backbone were described previously [45]. Stable expression sub-cell lines in PC-3, C4-2 and 293T cells were established with linearized constructs by Lipofactamine®2000 transfection (Life technologies) and single colons were selected in G418 with standard technology.

A 1250-kb fragment of DPYSL3v2 promoter sequence (−1275/−25 from the TSS) was cloned from human genome (NM_001387) and inserted into the up-stream of a Gaussia luciferase (GLuc) on a plasmid pEZX-LvPG02 by GeneCopoeia (Rockville, MD). A PCR-based three-step mutagenesis approach was utilized to generate the saRNA targeting site deletion mutants on the promoter, as illustrated in the supplementary Figure S3. The PCR primers were listed in supplemental Table S1. The mutated promoter fragments were ligated to the luciferase constructs with the unique EcoR*I* and BamH*I* sites and the final constructs were packaged into lentiviral particle using psPAX2/pMD2.G system as described above. The successful deletion was confirmed by direct sequencing (Genewiz, South Plainfield, NJ). Luciferase activity was measured with a Gaussia luciferase glow assay kit obtained from Life Technologies (Carlsbad CA).

### Total RNA extraction, quantitative RT-PCR and western blot analysis

Surgical tissue specimens from prostate cancers and the procedure for total RNAs extraction from cells and tissues were described in our recent publications [28, 46]. SYBR Green-based real-time PCR (RT-PCR) assays were conducted on the Bio-Rad iQ5 system. The primer pairs for individual genes were listed as follow: DPYSL3v1 forward 5′-GGT CCC GCG GCA GAA ATA C-3′; reverse 5′-GGC ATC GAA ATC CAG CGT CT-3′; DPYSL3v2 forward 5′-CGC CAC CAT GTC CTA CCA AG-3′; reverse 5′- GAC GAT TCT GCC TCC CTT GA-3′. PCR data analysis and primers for house-keeping gene S18 and KRT18 were described in our previous publications [28, 47].

Total cellular proteins were extracted from cells and tissues with radio-immuno-precipitation buffer (RIPA, Cell Signal) supplemented with protease inhibitor cocktails. Equal amount of proteins from each lysates was subjected to SDS-PAGE gels, electrophoresed, and transferred onto PVDF membrane. The membrane was incubated with primary antibody overnight at 4°C after blocking with 10% nonfat dried milk for 1 hour and then incubated with horseradish peroxidase-conjugated secondary antibody (Santa Cruz Biotech). Actin blot was used as an internal protein loading control in all blotting membranes.

### SBR cell growth, wound healing and Boyden Chamber assays

Cell growth was assessed using the sulforhodamine B assay as described [30, 48]. Wound healing assay was conducted to assess cell migration *in vitro* as described [49]. Briefly, sub-confluent monolayer cells in 24-well plates were scratched with a sterile 200 μl pipette tip to create a wound line in the middle of the well before transfection with saRNAs as indicated in the figures. Cell migration was monitored for 3 days.

Boyden Chamber transwells from Corning (Tewksbury, MA) were used to assess cell migration *in vitro* as described [50] with modifications. Briefly, 2 × 10^4^ cells were transfected with the saRNAs as indicated in 6-well plates and transferred into the upper chambers. Migrated cells in the lower chamber were stained with fluorescent dye Hoechst-33342 (final concentration at 5 μg/ml) and counted at 3 days after transfection.

### Animal experiments, tissue processing, immunohistochemistry and tissue luciferase detection

Athymic NCr-nu/nu male mice were obtained from NCI-Frederick and housed in accordance with the Institutional Animal Care and Use Committee (IACUC) procedures and guidelines. Human prostate cancer cells C4-2 expression luciferase reporter were trypsinized and resuspended in PBS. A total of 10^6^ cells were resuspended in RPMI-1640 and injected into the dorsal lobe of mouse prostate using a 1-ml disposable syringe. Xenograft tumor development was monitored every week with an IVIS living image system as described [14]. D-Luciferin potassium salt was purchased from GoldBiotech (St. Louis, MO). Once the tumor is established, animals were randomly divided into two groups (n = 5 mice per group) and received intraperitoneal injection of either A10-3.2-scramble or A10-3.2-saV2-9 saRNAs at a dose of 1.0 nmol/injection. Treatment was performed in 10 consecutive days. The mice were euthanized one day after the last injection. One hour before sacrifice, animals were received an intraperitoneal injection of 0.5 ml BrdU solution (10 mM, Roche Diagnostics, Indianapolis, IN) for *in situ* proliferation assay. Major organs including lung, liver, kidney, reginal lymph nodes and prostate, and xenograft tissues were excised. Half of the tissues were fixed with 4% paraformaldehyde for immunostaining and other half of the tissues were stored at −80°C for further experimentation.

Immunohistochemistry and H&E staining were conducted as described [51]. Briefly, paraffin embedded tissue sections were deparaffinized, rehydrated, followed by antigen retrieval and endogenous peroxidase blocking. The slides were incubated with primary antibody overnight at 4°C. Immunosignals were detected with DAKO LSABt System by following the manufacturer's manual. BrdU detection was conducted with a BrdU IHC kit (EMD Millipore, Billerica, MA). The picture was taken under a microscope (Nikon Inc., Melville, NY).

Luciferase activity in protein extracts from the whole lobe of right lung was assessed to evaluate micro-metastasis in the lung. Luciferase readings were normalized using total protein concentration.

### Statistical analysis

Statistical analysis was conducted using one-way ANOVA analysis with SPSS software (SPSS, Inc., Chicago, IL). A student T-test was performed to compare two groups. Tumor metastasis rate was compared with the Wilcoxon Rank-Sum Test. A p value of 0.05 was considered to indicate a significant difference. The mean and SEM from at least three repeated experiments are shown for all of the quantitative data.

## SUPPLEMENTARY FIGURES AND TABLE


