# Supplementary material for: Enhancing DPYSL3 gene expression via a promoter-targeted small activating RNA approach suppresses cancer cell motility and metastasis
Source: Oncotarget. 2016 Mar 23;7(16):22893–910. doi: 10.18632/oncotarget.8290 (PMC5008410; doi:10.18632/oncotarget.8290)
Supplement: Supplementary file 1 [file oncotarget-07-22893-s001.pdf]

SUPPLEMENTARY FIGURES AND TABLE

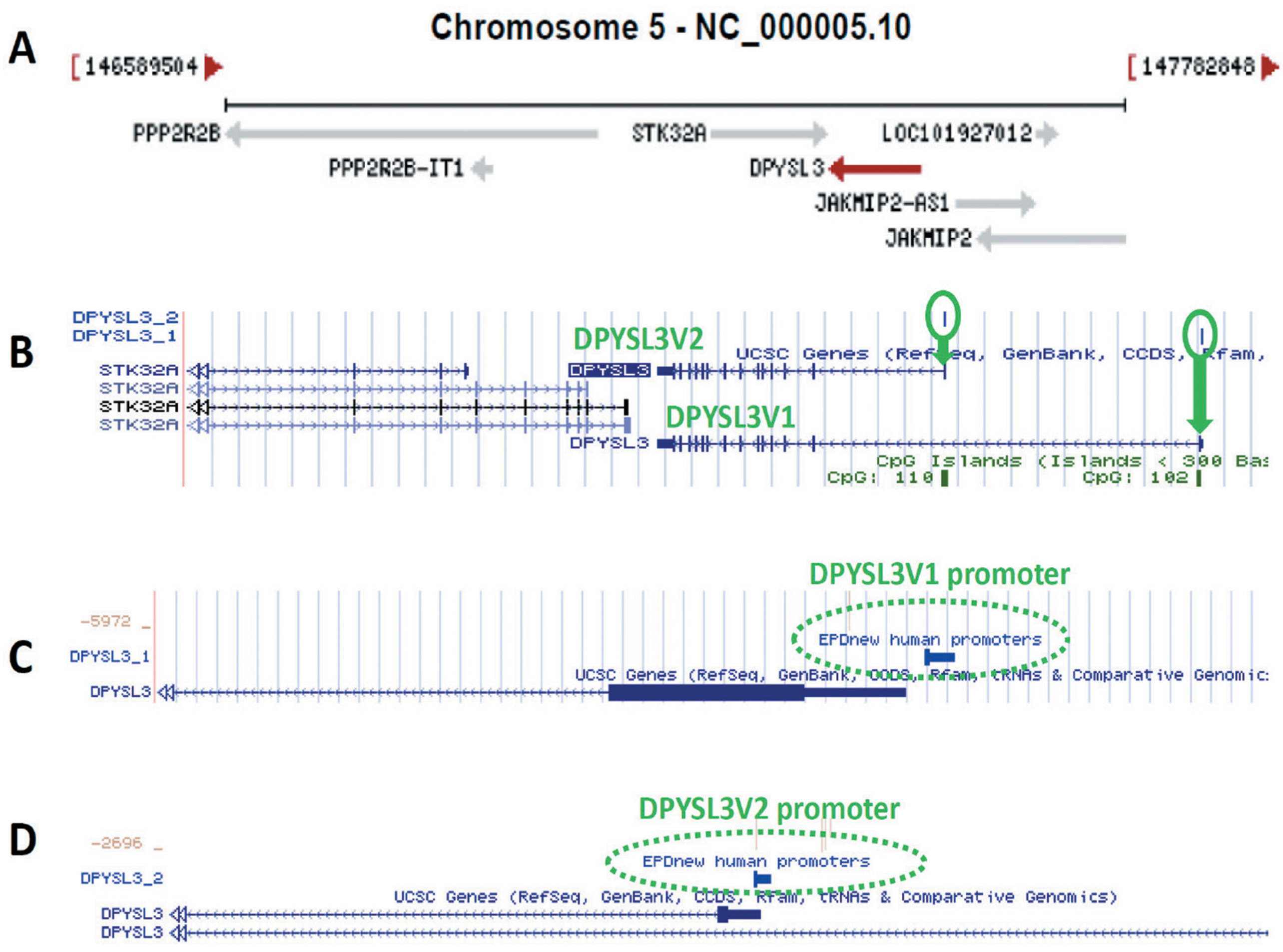

Supplementary Figure 1: Scheme of DPYSL3 gene location A. distinct prompters **B-D.** on chromosome 5. Data and images were extracted from UCSD genome browser.

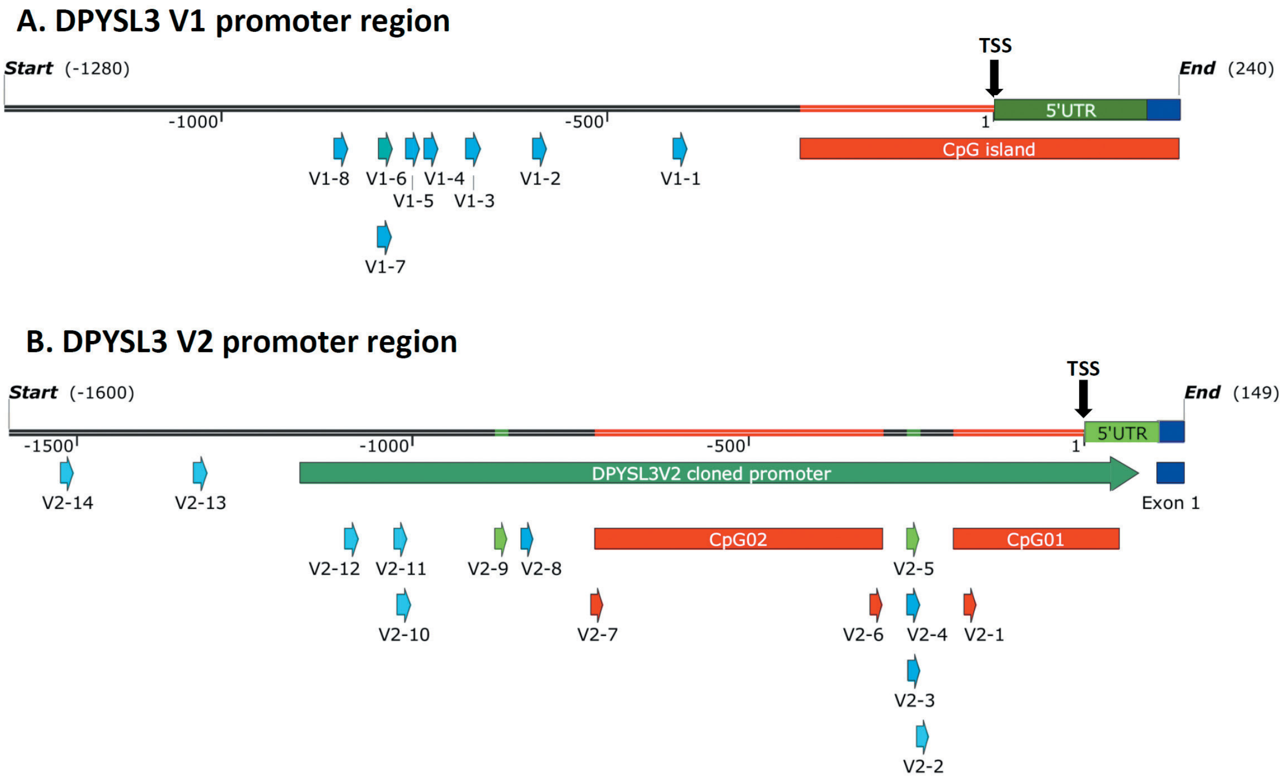

Supplementary Figure 2: Locations of DPYSL3v1 and v2-targeted saRNA sites (blue or light green arrows) and potential CpG islands (red bars) on the promoters within 1600 bp up-stream of the TSS site. Dark green arrow bar indicates the cloned DPYSLv2 promotor.

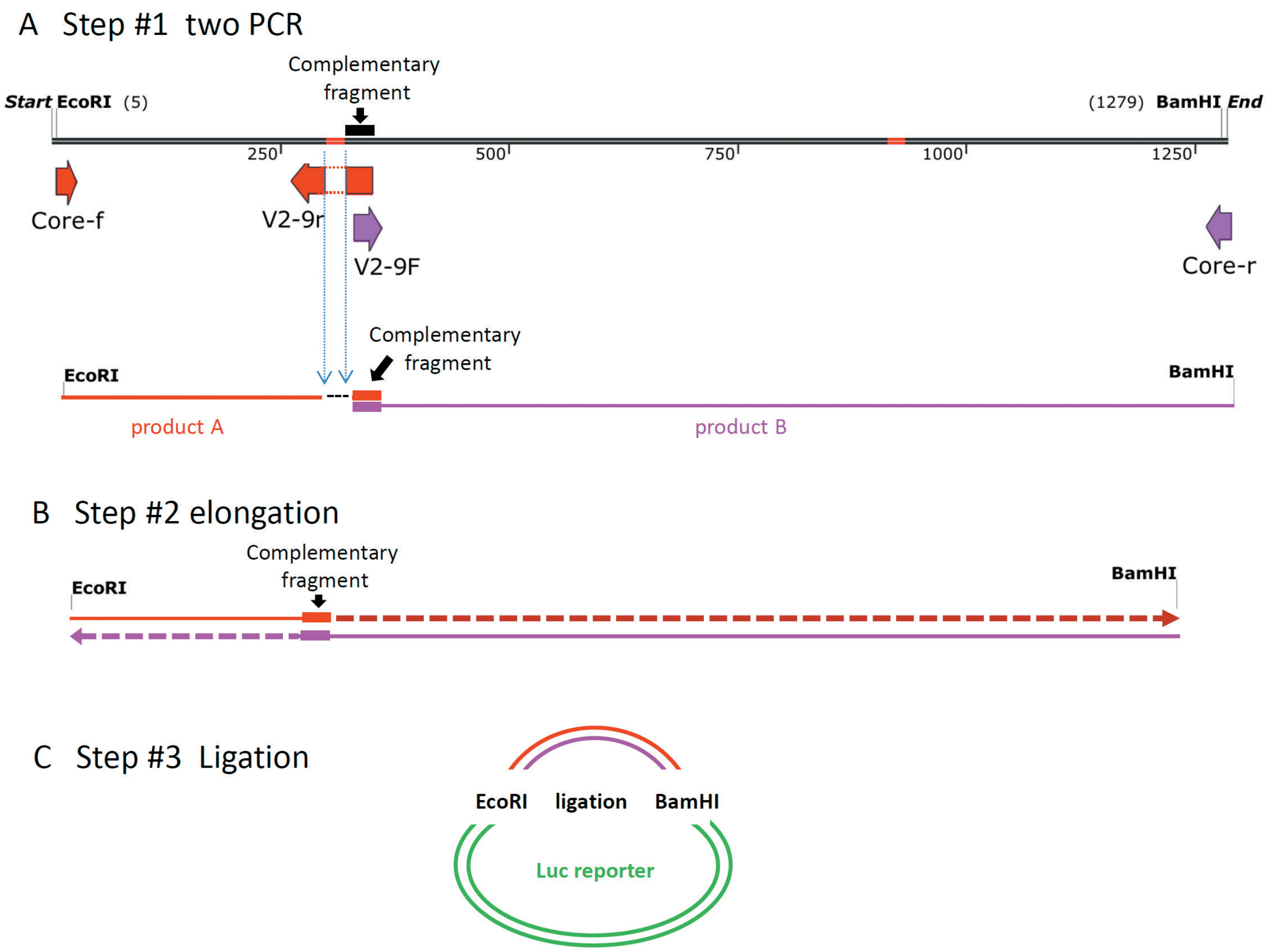

Supplementary Figure 3: Scheme of the three-step mutagenesis of the promoter with deleted saV2-9 and saV2-5 targeting sites. A. Step #1. PCR to generate two fragments (A & B) from the promoter and product A will loss the target site due to the specific design of the primer V2-9r. There is a complementary section on the A-3′ end and the B-5′ end as shown as a thicker red/pink bar. **B.** Step #2. Due to the complementary sequence on product A & B, purified product A and B was subjected to elongation reaction to produce a double-strand complementary DNA. **C.** Step #3. With the predefined unique enzyme sites on each ends, EcoRI and BamHI, the elongated DNA was ligated back to the original report plasmid backbone.

Supplementary Table 1: Primers for saRNA targeting sit deletion on DPYSL3v2 promoter

saRNA	PCR production	primer	sequence
***sa*V2-9**	A	Core-f	5'-CCGGAATTCAATTCACCCACAGTGG-3'
		V2-9-r	5'-CTGACTGAGTCTCAGAATCAAATCTGTGAGTAAAAAGTAGAAAACACCTG-3'
	B	V2-9-f	5'-TTTGATTCTGAGACTCAGTCAGGC-3'
		Core-r	5'-GCGGGATCCGAGCTCGGTACCA-3'
***sa*V2-5**	A	Core-f	5'-CCGGAATTCAATTCACCCACAGTGG-3'
		V2-5-r	5'-CACCCACTTTTTCTTTTTCTTTCTT-3'
	B	V2-5-f	5'-AGAAAAAGAAAAAGTGGGTGAATCAATAGGGATAAGAGAGAAGAGGG-3'
		Core-r	5'-GCGGGATCCGAGCTCGGTACCA-3'

Please note the complementary sequences were underlined in each primer.
